# Screening for rheumatoid arthritis-associated interstitial lung disease—a Delphi-based consensus statement

**DOI:** 10.1007/s00393-023-01464-w

**Published:** 2024-01-19

**Authors:** Klaus Hackner, Lisa Hütter, Holger Flick, Michael Grohs, Kastriot Kastrati, Hans Kiener, David Lang, Birgit Mosheimer-Feistritzer, Helmut Prosch, Eva Rath, Otmar Schindler, Florentine Moazedi-Fürst

**Affiliations:** 1 Division of Pneumology, University Hospital Krems, Karl Landsteiner University of Health Sciences, Krems, Austria; 2 2nd Department of Medicine, Hietzing Hospital, Wiener Gesundheitsverbund, Vienna, Austria; 3 Division of Pulmonology, Department of Internal Medicine, Medical University of Graz, Graz, Austria; 4 Center for Rehabilitation Engelsbad, Baden, Austria; 5 Department of Medicine III, Division of Rheumatology, Medical University of Vienna, Vienna, Austria; 6 Department of Internal Medicine 4—Pneumology, Kepler University Hospital, Johannes Kepler University, Linz, Austria; 7 Department of Internal Medicine II, Medical University of Innsbruck, Innsbruck, Austria; 8 Department of Internal Medicine, Hochzirl Hospital, Zirl, Austria; 9 Department of Biomedical Imaging and Image-Guided Therapy, Medical University of Vienna, Vienna, Austria; 10 1st Medical Department, Hanusch Hospital, Vienna, Austria; 11 Department of Internal and Respiratory Medicine, State Hospital Graz II, Gratwein, Austria; 12 Division of Rheumatology and Immunology, Department of Internal Medicine, Medical University Graz, Auenbruggerplatz 15, 8036 Graz, Austria

**Keywords:** Autoimmune diseases, Fibrosis, Antifibrotic agents, Risk factors, Mass screening, Autoimmunerkrankungen, Fibrose, Antifibrotika, Risikofaktoren, Massenscreening

## Abstract

**Objective:**

Rheumatoid arthritis-associated interstitial lung disease (RA-ILD) is a major driver of premature mortality in patients with rheumatoid arthritis (RA). Detection of RA-ILD is crucial but requires awareness among the treating physicians. To date, however, there is no international recommendation concerning screening for ILD in RA patients.

**Methods:**

After a systematic literature review, the modified Delphi technique in combination with the nominal group technique was used to provide a Delphi consensus statement elaborated by an expert panel of pneumonologists, rheumatologists, and a radiologist. Based on the available evidence, several clusters of questions were defined and discussed until consent was reached.

**Results:**

A screening algorithm for ILD in patients with RA based on clinical signs, respiratory symptoms, and risk factors has been developed. Further, the recommendations address diagnostic tools for RA-ILD and the follow-up of RA patients qualifying for ILD screening.

**Supplementary Information:**

The online version of this article (10.1007/s00393-023-01464-w) contains an additional table giving an overview of the questions provided to the expert panel and the individual group outcome.

## Abstract (de)

**Hintergrund:**

Die mit der rheumatoiden Arthritis (RA) assoziierte interstitielle Lungenerkrankung (RA-ILD) ist ein wesentlicher Faktor für die erhöhte Mortalität der Erkrankung. Die frühzeitige Diagnose ist für die Prognose bestimmend und liegt im Aufgabenbereich der behandelnden Spezialist:innen. Dennoch gibt es bislang keine internationalen Guidelines oder Screening-Empfehlungen für die RA-ILD.

**Methodik:**

Um ein Delphi-Konsensus-Statement zu erstellen, wurde eine Expert:innengruppe aus den Fachgebieten Pneumologie, Rheumatologie und Radiologie gebildet. Nach Analyse der aktuell verfügbaren Literatur erfolgten Fragerunden und Diskussionen, basierend auf der modifizierten Delphi-Methode in Kombination mit der nominalen Gruppenmethode.

**Ergebnis:**

Unter Berücksichtigung von klinischen Zeichen, Symptomen und Risikofaktoren wurde ein Algorithmus zur frühzeitigen Detektion einer ILD bei RA-Patient:innen ausgearbeitet. Darüber hinaus wurden Empfehlungen zu den Screening-Methoden sowie zum Follow-up bei RA-ILD formuliert.

Rheumatoid arthritis (RA) affects around 1% of the worldwide population. In RA, a systemic autoimmune reaction translates into chronic inflammation primarily at the synovium of diarthrodial joints. Pulmonary involvement, however, is frequent and associated with increased mortality [1].

Rheumatoid arthritis patients develop interstitial lung disease (ILD) nine times more often than the general population [2]. For RA patients the lifetime risk of developing clinically meaningful ILD is around 7–15% [3]. In more than three quarters of these patients, ILD occurs after the diagnosis of RA, usually within the first 5–10 years of the disease course [2, 4–7]. In less than 20% of the patients, however, ILD might be the first clinical finding of RA. Therefore, national and international guidelines, such as the latest German S1 guideline for diagnosis of ILD in adults, consistently recommend serological testing and clinical examination for autoimmune disease and RA in cases of newly detected ILD with unknown cause [8].

Besides cardiovascular disease and infections, ILD is a major driver of mortality in RA patients. In a population-based study, 1‑year mortality of RA-ILD was 13.9% as compared to 3.8% in RA patients without ILD [3]. In general, progressive fibrosing ILDs are associated with early mortality [9]. It is not uncommon, however, for RA-ILD to be diagnosed late in the course of the disease, since patients often experience minor or no symptoms in early stages [2, 4–7]. In fact, the prevalence of subclinical disease lies between 11.9 and 55.7% when entire RA populations are screened with a chest CT scan [10, 11].

Thus, screening RA patients for ILD seems promising to allow for an early diagnosis and to initiate therapy at an early stage of lung disease. Antifibrotic drugs have been shown to be effective in autoimmune progressive fibrosing ILDs [12]. Furthermore, diagnosis of RA-ILD often prompts a change in disease modifying antirheumatic drug (DMARD) therapy for RA patients. Lacking prospective drug trials on DMARDs in RA-ILD patients, treating physicians rely on observational data for individualized treatment. Since smoking is a known risk factor for both RA and lung disease in general, specific support for smoking cessation appears reasonable in any RA patient. Furthermore, granting low-level access to vaccinations against pulmonary pathogens for these patients appears reasonable as well.

The goal of early diagnosis of RA-ILD is a better outcome for the patients. Hence, early detection of RA-ILD requires awareness of the treating general physicians, rheumatologists, and pneumonologists. While there are recognized risk factors for the development of ILD among RA patients, an international recommendation for screening is still lacking. Some national attempts on this topic have been published [13, 14]. To date, however, there is no European Alliance of Associations for Rheumatology (EULAR) recommendation concerning screening for ILD in RA patients. The American College of Rheumatology (ACR) has announced the publication of a recommendation for late 2023. Of note, RA-ILD is not included as an extraarticular manifestation in the diagnostic criteria of RA.

To promote further awareness, the Austrian Societies of Rheumatology and Pneumology and the Austrian Society for Ultrasound in Medicine have convened a panel to elaborate a recommendation on the modalities of screening for RA-ILD. The objective of this Delphi consensus was to create a multidisciplinary proposal for screening criteria in RA patients that would enable early identification of patients with RA-ILD.

## Methods

For this Delphi consensus statement, a panel of experts was formed, including seven specialists in rheumatology and clinical immunology, four specialists in respiratory medicine/pneumonology, and one specialist in thoracic radiology. The expert panel was selected based on their experience in the topic and their scientific merit. Objectives of this statement were determined a priori. Therefore, the modified Delphi technique in combination with the nominal group technique was used and consisted of five steps [15, 16]:

### Step 1

In the first session, the experts agreed on a moderator of the Delphi process (KH). Initially, literature databases were to be searched to identify relevant publications on epidemiology, diagnosis, monitoring methods, and screening for RA-ILD. All literature and background information were provided to the expert panel. Then the voting process was defined and the maximum number of rounds was determined. Polling was set to be anonymous via email and only the moderator was informed about the individual votes. The definition of consensus was predetermined and set to a level of agreement > 60% and consent with a median value of ≥ 3 on the four-point scale (fully agree, partly agree, partly disagree, fully disagree, and an option for abstention), with a lower interquartile range (IQR) of ≥ 3. Group responses were to be provided to all participants after each voting round.

### Step 2

The second step was concluded by describing five clusters for voting questions by the expert panel. The first cluster of questions was dedicated to symptoms and awareness of RA-ILD (I). The second cluster of questions was focused on patients with RA who present with respiratory symptoms suggesting RA-ILD (e.g., persistent cough and/or dyspnea on exertion; II). The third cluster was dedicated to RA patients without symptoms of RA-ILD but with risk factors (III). The most suitable diagnostic procedures for RA-ILD baseline examinations were subject to debate in the fourth cluster (IV). Cluster five was dedicated to the most suitable follow-up examination for patients referred for regular RA-ILD screening with normal baseline findings (V). Questions for each cluster were drafted based on the literature review.

### Step 3

After completing the anonymous voting, the group results of the voting were discussed in the panel. When no consent was reached, the expert panel defined another question based on the comprehensive discussion to resolve the discussion.

### Step 4

Another anonymous voting round was completed, followed by a discussion of the results and definition of further questions in case of insufficient consent on a question.

### Step 5

In a final anonymous voting round, the remaining questions were answered with consent, and consensual recommendations and an illustrative flowchart were created based on the voting results.

## Results

### Cluster I

The panel found consent and full agreement (100%) for the fact that a clinically significant RA-ILD is present in 7–15% of RA patients and is an important cause of mortality and loss of quality of life, and for the fact that preclinical RA-ILD poses an important factor in therapeutic decision-making in RA. Patients with RA should be asked for respiratory symptoms such as persistent cough and dyspnea on exertion, and undergo auscultation regularly (91% agreement). No consent was found for the additional use of a questionnaire to screen for respiratory symptoms of RA-ILD, since there is currently no approved questionnaire for this condition available. Questionnaires such as the Modified Medical Research Council, St. Georges Respiratory Questionnaire and the American College of Chest Physicians (ACCP) ILD questionnaire, etc., were designed for different purposes and do not specifically address the symptoms of RA-ILD.

### Cluster II

The panel found consensus and agreement that patients who present with respiratory symptoms suggesting RA-ILD should be further examined with pulmonary function testing consisting of body plethysmography, spirometry, and diffusing capacity of the lungs for carbon monoxide (DLCO) testing as well as radiological assessment. High-resolution CT (HR-CT) without intravenous contrast is the radiological examination of choice for the detection of ILD. Chest radiographs have a low diagnostic yield for ILD and were considered an insufficient diagnostic tool in screening for RA-ILD. However, for differential diagnosis of general respiratory symptoms, e.g., diseases such as acute bacterial pneumonia, chest radiographs are a valid option.

### Cluster III

Full consensus was found concerning the currently known risk factors for RA-ILD: smoking, elevated cyclic citrullinated peptide (CCP) antibodies and rheumatoid factor (RF), male sex, high disease activity, advanced age, and family history of RA-ILD. However, after two anonymous question rounds with subsequent discussions of the group vote, no consent was found on their individual value or regarding which combination of risk factors should prompt regular RA-ILD screening. The currently available data did not enable the panel to make an evidence-based statement. Therefore, until further evidence is provided, RA patients without respiratory symptoms but with known risk factor(s) for RA-ILD should be included into RA-ILD screening at the discretion of the treating physician on the basis of a case-by-case decision, which was consented and fully agreed upon (100%) by the panel in the third question round.

### Cluster IV

Concerning examinations for RA patients without respiratory symptoms but with risk factors, the panel consented on pulmonary function testing (body plethysmography, spirometry), DLCO testing, and non-contrast HR-CT at baseline. In case of results suggesting RA-ILD at the baseline examinations, further management of the patient should be discussed in a multidisciplinary board (level of agreement 100%; full consent).

### Cluster V

**Fig. 1:**
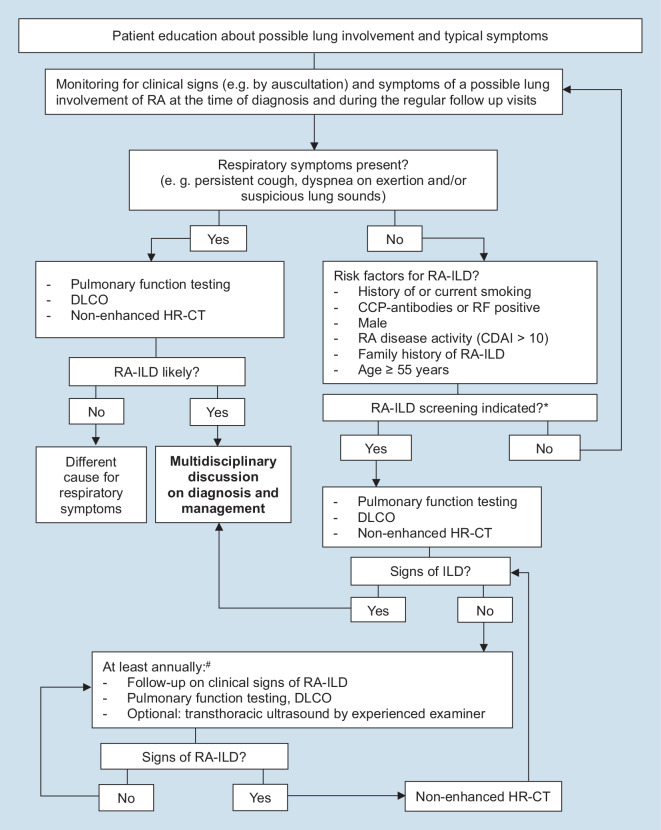
Suggested screening algorithm for rheumatoid arthritis-associated interstitial lung disease (*RA-ILD*) in incident patients with rheumatoid arthritis and follow-up after initial screening. *RA* rheumatoid arthritis, *DLCO* diffusion limitation for carbon monoxide, *anti-CCP* cyclic citrullinated peptide antibodies, *RF* rheumatoid factor, *HR-CT* high-resolution computed tomography, *CDAI* Clinical Disease Activity Index. *Asterisk* Quantity and significance of existing risk factors to trigger continuous RA-ILD screening cannot be given at the moment, and these are therefore at the discretion of the treating physician on a case-by-case decision. Rhombus Necessity of regular non-contrast HR-CT and diagnostic interval of examinations is at the discretion of the attending physician on the basis of a case-by-case decision

Concerning the most suitable follow-up examination for patients referred for regular RA-ILD screening with normal baseline findings, the panel agreed that regular HR-CT was not the most appropriate follow-up examination, but rather that follow-up on pulmonary function tests including DLCO and monitoring of clinical signs of RA-ILD should be performed. As no initial consent on the time period of follow-up examinations was found, the panel agreed that screening should be performed at least annually for patients with initially inconspicuous RA-ILD. Evidence on the repeated use of HR-CT was not regarded as sufficient; therefore, the panel voted that the decision to perform regular non-contrast HR-CT periodically in RA patients without respiratory symptoms but with known risk factor(s) for RA-ILD can be done at the discretion of the attending physician on the basis of a case-by-case decision. Furthermore, optional transthoracic ultrasound performed by an experienced examiner found consent and agreement in this context. An overview of the questions provided to the panel and the individual group outcome (level of agreement; consent) is provided as online supplemental material. Finally, the outcome of each cluster in the Delphi consensus was transcribed into a combined algorithm (Fig. 1).

## Discussion

The aim of this Delphi consensus statement was to develop a stepwise screening algorithm for RA-ILD and to raise awareness among physicians regularly taking care of RA patients. Given the high mortality of RA-ILD and the availability of new therapeutic options such as nintedanib for the progressive pulmonary fibrosis phenotype of RA-ILD, early diagnosis of ILD is essential, and can be achieved by screening [12]. However, the diagnostic methods and the timing of screening are still subject to debate. Monitoring of clinical signs (e.g., by auscultation) of possible lung involvement of RA at the time of diagnosis and during the regular follow-up visits was fully supported by the expert panel, as was the necessity of further investigations in case of suspicious respiratory symptoms (i.e., persistent cough or dyspnea on exertion). Further steps suggested are lung function testing with DLCO testing, body plethysmography, and HR-CT of the lung. DLCO has become an important standard in pulmonary function testing, next to forced vital capacity or total lung capacity [17]. It is a strong and independent predictor of diffusion limitation and often the first sign of lung fibrosis. Particular interest should be given to lung auscultation, since Velcro crackles have been found to be a key finding in early fibrotic lung disease [18, 19]. It was discussed that combined measurements of lung volumes including diffusion capacity and the HR-CT should be used rather than the individual examinations alone. In case of non-corresponding results of lung function testing or HR-CT, a multidisciplinary discussion is mandatory to evaluate the presence of RA-ILD or differential diagnoses. This must be emphasized, since patients with RA may also suffer from coexisting ILD other than RA-ILD, with respective treatment indications. It is important to highlight that a multidisciplinary discussion should at least include specialists in pneumonology, rheumatology, and radiology, each with expertise in ILD [20]. Using HR-CT to detect RA-ILD appears to be the best approach in terms of radiographic imaging. However, RA-ILD patterns vary, and may present as usual interstitial pneumonia (UIP), probable for UIP, non-specific interstitial pneumonia, organizing pneumonia, mixed patterns, unclassifiable patterns, and others [21]. To avoid radiation exposure, the panel gave no general recommendation to screen all newly diagnosed RA patients but rather to focus on a population at higher risk. Therefore, the expert panel recommended evaluating risk factors for RA-ILD in each patient with RA, even in the absence of respiratory symptoms. Well established and known risk factors are current or former smoking (especially more than 25 packyears), age, male sex, high disease activity (e.g., by a Clinical Disease Activity Index [CDAI] score > 10), and a family history of and genetic predisposition for RA-ILD [1, 19, 22–28]. A particular threshold for age as a risk factor has not yet been approved, but different studies found clinically significant RA-ILD from > 55 years upwards [29–34].

Nevertheless, it has not yet been resolved how individual risk factors should be graded and translated into a clinically confident situation to further investigate for RA-ILD. Therefore, the panel concluded that the quantity and significance of existing risk factors for initiating continuous RA-ILD screening lies at the discretion of the attending physician on the basis of a case-by-case decision. From the panel’s perspective, there is currently not enough evidence to support any specific scoring or weighting of risk factors. These recommendations will be updated as soon as new and profound evidence is published about the classification of known RA-ILD risk factors.

In this context it must be mentioned that a Spanish recommendation for ILD screening in RA patients was published after the Delphi consensus had been designed [35, 36]. In this Delphi consensus, risk factors were weighted and an arbitrary cut-off of 5 points was chosen for conducting ILD screening in RA patients. However, no confirmation of this score has been published so far, thus making it an expert recommendation based on experience rather than evidence. The important, yet unanswered question is whether or not more risk factors will forecast a higher likelihood of developing RA-ILD.

If a patient qualifies for RA-ILD screening, a baseline pulmonary function test (including DLCO testing) and HR-CT should be performed, as suggested by the panel. For regular follow-up, we suggest looking for clinical sings of RA-ILD and to perform pulmonary function testing including DLCO on an annual basis. Another option is transthoracic ultrasound with evaluation of B lines. This technique has emerged over the past years as an alternative method to evaluate ILD in patients with connective tissue diseases, particularly systemic sclerosis [37, 38], but also RA-ILD [39, 40]. However, transthoracic ultrasound is still rarely applied in the ambulant care of RA patients and might gain increased popularity in the future. The regular implementation of HR-CT was discussed critically in this particular situation, especially in patients without symptoms. Ionizing radiation is a general matter of concern, as it is a recognized cause of cancer [41]; however, there is also currently not enough evidence to suggest that screening using HR-CT could generate a prognostic benefit for the patient. Thus, there is currently no positive benefit–risk profile available to support regular HR-CT in patients with RA and risk factors for RA-ILD but without respiratory symptoms. Therefore, the necessity of regular HR-CT and the interval of diagnostic examinations are at the discretion of the attending physician on the basis of a case-by-case decision. If new evidence is published, this recommendation will be updated.

In recent years, different studies have reported a *MUC5B* promoter variant rs5705950 as a rare but significant risk factor for developing RA-ILD, even in the absence of respiratory symptoms [30, 33, 42]. Juge et al. also proposed a risk score to evaluate for subclinical RA-ILD, including sex, age at RA onset, RA disease activity using the Disease Activity Score-28 for Rheumatoid Arthritis with erythrocyte sedimentation rate (DAS28-ESR), and the abovementioned *MUC5B* rs35705950 genetic variant. However, the predictive value of this risk score still needs validation in prospective studies [30]. A future perspective will be the announced European Respiratory Society (ERS)/EULAR connective tissue disease (CTD)-associated ILD management guidelines, which will include screening.

Some limitations and weaknesses of this RA-ILD screening must be mentioned, especially the fact that the current evidence did not allow the expert panel to establish a risk score or a weighting of known risk factors for RA-ILD. Until additional data become available, the task of selecting RA patients based on risk factors for RA-ILD must be left in the hands of the caring physicians, thus making it open to interpretation. However, interdisciplinary awareness for RA-ILD as well as its proper tools for (differential) diagnosis are the major goals of our statement.

## Conclusion

This Delphi-based screening strategy was developed for early detection and ongoing surveillance of RA-ILD by a multidisciplinary team of pneumonologists, rheumatologists, and radiologists. The presented algorithm was designed to help carers of patients with RA to identify those at risk for RA-ILD and choose an applicable screening strategy for these patients. Furthermore, disease awareness for RA-ILD among carers and patients is of utmost importance. Since risk factors for RA-ILD are well known but cannot be weighted at the moment, an updated version of this recommendation will be necessary when further evidence on this topic is published.

### Supplementary Information

Additional Table 1: Overview of the questions provided to the expert panel and the individual group outcome

## Declarations

For this article no studies with human participants or animals were performed by any of the authors. All studies mentioned were in accordance with the ethical standards indicated in each case.
