# Supplementary material for: Screening for rheumatoid arthritis-associated interstitial lung disease—a Delphi-based consensus statement
Source: Z Rheumatol. 2024 Jan 19;83(2):160–8. doi: 10.1007/s00393-023-01464-w (PMC10902070; doi:10.1007/s00393-023-01464-w)
Supplement: Supplementary file 1 — Additional Table 1: Overview of the questions provided to the expert panel and the individual group outcome [file 393_2023_1464_MOESM1_ESM.pdf]

Supplemental online material to:

Screening for Rheumatoid Arthritis-Interstitial Lung Disease – a Delphi-based Consensus Statement

K. Hackner, L. Hütter, H. Flick et al.; Zeitschrift für Rheumatologie

Overview of the questions provided to the expert panel and the individual group outcome

Voting round	Cluster	Question	Responses n (% of panel)	Level of agreement*	Consent* Median (IQR)
Round 1	I	A clinically significant RA-ILD is present in 8-15% of RA patients and is an important cause of mortality and quality of life.	11 (92)	100%	4 (4-4)
		Preclinical RA-ILDs poses an important factor in the therapeutic decision in RA.	11 (92)	100%	4 (4-4)
		Patients with RA should be asked for respiratory symptoms (e. g. persistent cough, dyspnea on exertion) regularly.	11 (92)	100%	4 (3-4)
		Patients with RA should undergo regular auscultation.	11 (92)	91%	3 (3-4)
		Patients with RA should be informed about a possible lung involvement of the rheumatic disease.	11 (92)	91%	4 (3-4)
		Additional question: A questionnaire should be used to screen for respiratory symptoms in patients with RA.	7 (59)	57%	3 (2-3)
	II	Patients with RA and respiratory symptoms (e. g. persistent cough, dyspnea on exertion) should undergo pulmonary function testing and DLCO testing prior to radiological examination.	11 (92)	73%	4 (3-4)
		The primary radiological examination in patients with RA and respiratory symptoms (e.g. persistent cough, dyspnea on exertion) should be a non-contrast HR-CT.	12 (100)	100%	4 (4-4)
	III	Risk factors for RA-ILD are: h/o or current smoking, CCP-antibodies, RF, male sex, increased disease activity (CDAI >10), family history of RA-ILD.	12 (100)	100%	4 (4-4)
		RA patients without respiratory symptoms suggesting RA-ILD should receive further diagnostic examinations for possible RA-ILD if one known risk factor for RA-ILD is present.	12 (100)	58%	3 (2-3)
		RA patients without respiratory symptoms suggesting RA-ILD should receive further diagnostic examinations for possible RA-ILD when ≥ 2 known risk factors for RA-ILD are present.	11 (92)	91%	3 (3-4)
		RA patients without respiratory symptoms suggesting RA-ILD should receive further diagnostic examinations for possible RA-ILD when a history of or current smoking status is present.	12 (100)	83%	3 (3-4)
		In case of no identified risk factors for RA-ILD in RA patients without respiratory symptoms, age ≥ 50 years should lead to further diagnostic examinations for possible RA-ILD	10 (83)	20%	1.5 (1-2.25)

	IV	RA patients without respiratory symptoms of RA-ILD, but with risk factor(s) for RA-ILD should receive HR-CT as the primary radiological examination for RA-ILD.	10 (83)	70%	3.5 (2-4)
		RA patients without respiratory symptoms of RA-ILD, but with risk factor(s) for RA-ILD should receive pulmonary function testing, including DLCO, and chest radiography to exclude RA-ILD.	11 (92)	63%	3 (2-3)
		In RA patients without respiratory symptoms of RA-ILD, but with risk factor(s) for RA-ILD, normal pulmonary function tests, including DLCO, and normal chest radiography enable to exclude RA-ILD at that time.	11 (92)	36%	2 (2-3)
		In RA patients without respiratory symptoms of RA-ILD, but with risk factor(s) for RA-ILD, a normal transthoracic ultrasound, performed by an experienced examiner, enables exclusion of RA-ILD at that time.	9 (75)	66%	3 (2-3)
	V	In RA patients without respiratory symptoms of RA-ILD, but with risk factor(s) for RA-ILD, and without evidence for RA-ILD in the further diagnostic examination(s), follow-up should be performed with regular HR-CT.	10 (83)	0%	1 (1-2)
		In RA patients without respiratory symptoms of RA-ILD, but with risk factor(s) for RA-ILD, and without evidence for RA-ILD in the further diagnostic examination(s), follow-up should be performed with regular pulmonary function testing, including DLCO, and chest radiography.	12 (100)	66%	3 (2-3)
		In RA patients without respiratory symptoms of RA-ILD, but with risk factor(s) for RA-ILD, and without evidence for RA-ILD in the further diagnostic examination(s), follow-up should be performed with regular transthoracic ultrasound, performed by an experienced examiner.	10 (83)	100%	3 (3-4)
Round 2	I	In patients with RA, monitoring of clinical signs (e.g. by auscultation) of a possible RA-ILD at the time of diagnosis and during the regular RA follow-up visits should be performed.	11 (92)	100%	4 (4-4)
	II	An HR-CT suggesting RA-ILD should lead to a multidisciplinary discussion of the patient’s further management.	12 (100)	100%	4 (4-4)
	III	Known risk factors for RA-ILD (h/o or current smoking, CCP-antibodies, RF, male sex, increased disease activity (CDAI >10), family history of RA-ILD, age ≥55 years) pose a major role for the decision to screen RA patients without respiratory symptoms for RA-ILD.	12 (100)	100%	4 (4-4)
		Additional question: The number of present risk factors is important for this decision.	8 (67)	75%	3 (1-4)
		In RA patients without respiratory symptoms but with risk factor(s) for RA-ILD, who were selected to be screened for RA-ILD, HR-CT and pulmonary function testing including DLCO, should be performed.	12 (100)	100%	4 (3-4)
	V	Follow-up examinations for RA-ILD screening should include pulmonary function testing including DLCO.	11 (92)	82%	4 (3-4)
		Follow-up examinations for RA-ILD screening should include HR-CT at least after 5 years, even in case of normal pulmonary function testings and DLCO.	11 (92)	63%	3 (1-4)
		Follow-up examinations for RA-ILD screening should include pulmonary function testing including DLCO and optional may include transthoracic ultrasound by an experienced examiner.	12 (100)	100%	4 (4-4)

Round 3	III	Currently, there is not enough evidence on the required quantity and the intrinsic value of known RA-ILD risk factors (including age), to provide an objective statement on the initiation of RA-ILD screening. Therefore, until further evidence is provided, RA patients without respiratory symptoms but with known risk factor(s) for RA-ILD should be included into RA-ILD screening at the discretion of the treating physician on a case-by-case decision.	12 (100)	100%	4 (4-4)
	V	Currently, there is not enough evidence on the use and intervals of HR-CT for regular RA-ILD screening to provide an objective statement. Therefore, until further evidence is provided, the decision to perform regular HR-CT (additionally to clinical follow up, pulmonary function testing, DLCO, and optional pleurasonography) in RA patients without respiratory symptoms but with known risk factor(s) for RA-ILD is at the discretion of the treating physician on a case-by-case decision.	12 (100)	100%	4 (4-4)

* The definition of consensus was predetermined and set to a level of agreement ≥70% AND consent with a median value of ≥3 on the 4-point scale (fully agree=4, partly agree=3, partly disagree=2, fully disagree=1; abstention) with a lower interquartile range ≥3.
